# Molecular Markers and Cotton Genetic Improvement: Current Status and Future Prospects

**DOI:** 10.1155/2014/607091

**Published:** 2014-10-23

**Authors:** Waqas Malik, Javaria Ashraf, Muhammad Zaffar Iqbal, Asif Ali Khan, Abdul Qayyum, Muhammad Ali Abid, Etrat Noor, Muhammad Qadir Ahmad, Ghulam Hasan Abbasi

**Affiliations:** ^1^Department of Plant Breeding and Genetics, Faculty of Agricultural Sciences and Technology, Bahauddin Zakariya University, Multan, Pakistan; ^2^Agricultural Biotechnology Research Institute, Ayub Agriculture Research Institute, Faisalabad, Pakistan; ^3^Department of Plant Breeding and Genetics, University of Agriculture, Faisalabad, Pakistan; ^4^University College of Agriculture and Environmental Sciences, The Islamia University of Bahawalpur, Bahawalpur, Pakistan

## Abstract

Narrow genetic base and complex allotetraploid genome of cotton (*Gossypium hirsutum* L.) is stimulating efforts to avail required polymorphism for marker based breeding. The availability of draft genome sequence of *G. raimondii* and *G. arboreum* and next generation sequencing (NGS) technologies facilitated the development of high-throughput marker technologies in cotton. The concepts of genetic diversity, QTL mapping, and marker assisted selection (MAS) are evolving into more efficient concepts of linkage disequilibrium, association mapping, and genomic selection, respectively. The objective of the current review is to analyze the pace of evolution in the molecular marker technologies in cotton during the last ten years into the following four areas: (i) comparative analysis of low- and high-throughput marker technologies available in cotton, (ii) genetic diversity in the available wild and improved gene pools of cotton, (iii) identification of the genomic regions within cotton genome underlying economic traits, and (iv) marker based selection methodologies. Moreover, the applications of marker technologies to enhance the breeding efficiency in cotton are also summarized. Aforementioned genomic technologies and the integration of several other omics resources are expected to enhance the cotton productivity and meet the global fiber quantity and quality demands.

## 1. Introduction

Cotton (*Gossypium* spp.) is considered as the foremost natural fiber and oil source worldwide, with an estimated production and utilization of ~115 million bales [1]. It is indigenous to tropical and subtropical regions and being cultivated on every continent excluding Antarctica [2]. The economic impact of the cotton industry throughout the world is about ~$500 billion per year [3]. Despite its economic value, cotton has also an outstanding model system for studying cell elongation, polyploidization, cellulose, and biosynthesis of cell wall [4–6], because it is the only familiar plant that yields single-celled fiber [4].

Cotton belongs to genus* Gossypium* and family Malvaceae. The genus* Gossypium* has 45 diploid and 5 allotetraploid species and occur in semiarid and arid areas of Africa, Central and South America, Galapagos, Indian subcontinent, Australia, Arabia, and Hawaii [7]. These 50 species are allotted to 8 diploid genomes (A–G and K) [8–10]. The A, B, E, and F genomes naturally occur in Africa and Asia, while the D genome is indigenous to the America [11]. A third diploid clade, containing C, G, and K, is found in Australia [12]. Currently cotton has only 4 cultivated species, two tetraploid species [*G. hirsutum *L. (AADD) and* G. barbadense *L. (AADD), (2*n* = 4*x* = 52)] and two diploid species [*G. arboreum *L. (A_2_A_2_), and* G. herbaceum *L. (A_1_A_1_), (2*n* = 2*x* = 26] [13]. Cultivated tetraploid cotton evolved about 1-2 million years ago through hybridization of an A genome donor species (*G. herbaceum *and* G. arboreum*) with a D genome (*G. raimondii *and* G. gossipioides*) followed by polyploidization [14–16]. The progenitor allotetraploid “AD” diverged and gives rise to “AD” tetraploid species (*G. hirsutum *L. and* G. barbadense *L.) [17].* G. hirsutum *(Upland cotton or Mexican cotton) contributes 90%;* G. barbadense* (Sea Island cotton or Egyptian cotton) produces 8% [18, 19],* G. herbaceum* (Levant cotton) and* G. arboreum* (Tree cotton) together provide 2% of the world's cotton [20].

Tetraploid genome of cotton is relatively large and contains about 2200–3000 Mb of DNA [21, 22]. The intraspecific DNA polymorphism is low in this species [23, 24], which makes it a challenging crop for development of molecular markers. There is an undeniable need for highly polymorphic molecular markers if progress in plant breeding is to be made using marker-assisted breeding programs. Many extraordinary reviews have been written about the different classes of molecular markers used in plants and their application in construction of linkage map, QTL analysis and marker-assisted selection [25–27]. The objectives of this review are as follows: (i) analysis of the evolution of molecular marker technologies in cotton genetics, (ii) genetic diversity in the wild and cultivated cotton gene pools, and (iii) overview of QTL mapping and marker assisted selection activities in cotton.

## 2. Overview of Molecular Marker Technologies in Cotton

Molecular markers are the firm landmarks in the genome of an organism rather than the normal genes because mostly they do not have the biological impacts and may or may not relate with phenotypic expression of a trait [26]. The development of the DNA markers is simple due to the availability of large scale genomic database [28]. In plant breeding, these markers are very helpful in recognition, characterization, identification of genetic variations, marker assisted selection (MAS), linkage mapping, and genomic fingerprinting [29], to remove linkage drag in backcrossing and to identify the traits which are not easy to measure by visual observation [30]. Molecular marker technologies can be classified into hybridization based, PCR based, and sequenced based markers on the basis of their working mechanism. Among these, PCR-based markers, that is, random amplified polymorphic DNA (RAPD) [23, 31, 32], amplified fragment length polymorphism (AFLP) [17, 33], simple sequence repeats microsatellites (SSRs) [34, 35], and inter simple sequence repeats (ISSRs) [36], represent the major class of markers in cotton genomics due to their high utility and exploitation. The comparison of different aspects of generally used molecular markers is given in Table 1 and brief description of these three classes of molecular markers is described below with special reference to cotton genetic.

### 2.1. Hybridization Based DNA Markers

Restriction fragment length polymorphism (RFLP) markers reveal the differences among individuals by variation in the size of DNA fragments produced by restriction enzymes [27]. These markers enabled DNA variations to be tested as substitutions of a single base in the recognition sequence of a restriction enzyme altered the length of resultant restriction fragments [37]. In this method cDNA or synthetic oligonucleotides are used as probes and DNA profiles are observed by hybridizing the restricted DNA fragment to a labeled probe (labeled with radioisotope). RFLPs can be used to examine the association between the closely related taxa, for study of introgression and gene flow between crops and weeds [38]. In various species of cotton, RFLP markers have been used to study the population genetics, evolution, and phylogenetic relationships [39]. Various reports are published on genetic mapping of cotton using RFLPs (restriction fragment length polymorphism) [40–42], and it was reported that in cotton 64% RFLPs are codominant in nature [43]. Genetic diversity in upland cotton has also been examined using RFLP markers [38]. Molecular map of the cotton genome was first constructed using 705 RFLP loci and partitioned into 41 linkage groups [43]. The utility of RFLP markers in marker assisted selection (MAS) is reported and RFLP linked to resistance allele for pathogen of bacterial blight was validated [44]. RFLP markers are very complex and time and cost intensive technique which restricted it use, leading to development of less complicated techniques known as PCR base markers [26].

### 2.2. PCR Based Markers

PCR (polymerase chain reaction) is used for replication of small amount of DNA enzymatically without using the living organism. DNA polymerase, such as* Taq* polymerase, reads and synthesizes a new strand in 5′-3′ direction using deoxynucleotide triphosphates (dNTP's). It can not only amplify small quantity of DNA, but degraded sources of DNA can also be amplified [45]. The reaction of PCR consists of many cycles of denaturation, annealing, and extension. Then, the PCR product can be visualized on agarose or polyacrylamide gels. PCR-based technology has been utilized widely in analysis of genetic diversity and recognition of DNA markers. Due to the simplicity and high chances of success in PCR, many approaches for production of PCR based molecular markers were described.

#### 2.2.1. Random Amplified Polymorphic DNA, RAPD

RAPD is an old PCR based technique that infers DNA polymorphisms due to deletions or reorganization between the obligating sites of oligonucleotide primer in the genome [46]. In RAPDs, DNA fragments are amplified by the PCR reaction using random primers (usually of 10 bp) [47]. The sequence of the RAPD primers must fulfill the following criteria: (i) minimum 40% GC contents and (ii) absence of palindromic sequence [46]. A discrete DNA product is produced if these priming sites are within an amplifiable range of each other.

RAPD techniques have been used for many purposes including assessment of genetic variations in population [23, 48], DNA fingerprinting [49], and determining the relationship between the genotypes of different and same species [50]. In cotton RAPDs were used to distinguish the cotton varieties resistant to jassids, aphids, and mites [51]. RAPD marker (R-6592) for the male sterility gene has been identified in cotton [52]. RAPD techniques are also used to evaluate the genetic relationship among cotton genotypes [53], to identify the QTLs for stomatal conductance [54], and to construct linkage mapping in cotton.

#### 2.2.2. Inter Simple Sequence Repeats, ISSR

In ISSRs, DNA fragments are amplified which present between two identical SSRs directed in contrary directions [47]. It allows the detection of polymorphism in inter SSR loci using primer (16–25 bp long) complimentary to a single SSR and anneal at either the 3′ or 5′ end [47], that can be di, tri, tetra or pentanucleotide [36]. The ISSR primers are commonly anchored at 3′ or 5′ end with 1 to 4 bases stretched into the flanking regions. The primers anchored at 3′ end produce more obvious bands as compared to anchored at 5′ end [36]. The technique of ISSR markers combines many benefits of AFLPs and SSRs with universality of RAPDs [55]. Generally the sequence of ISSR primers is larger as compare to RAPD primers, allowing higher annealing temperature which outcomes greater reproducibility of bands than RAPDs [36, 56]. Amplification of ISSRs also revealed larger fragments number per primer than RAPDs [57]. Many earlier studies reported that ISSR markers were more informative than RAPDs for genetic diversity evaluation in different crop species [58, 59].

The applications of ISSRs for different purposes depend on the diversity and frequencies of SSR within the particular genomes [60]. It is quickly being utilized by the research community in different areas of plant improvement, that is, in gene tagging, analysis of genetic diversity, and estimation of SSR motif [61–63].

#### 2.2.3. Amplified Fragment Length Polymorphism, AFLP

AFLP markers were developed to overcome the problem of reproducibility connected to RAPDs [64]. This technique detects large number of loci in a single reaction of PCR [64, 65] and discovers large number of polymorphism dispersed across the genome [66]. In AFLP assays amplicon numbers are depend on (i) number of selective nucleotides in the primer, (ii) selective nucleotide motif, (iii) GC content, and (iv) physical genome size [26]. AFLP is an effective tool for the observation of genetic diversity [67], fingerprinting studies, and tagging of agronomic, seed, and fiber quality traits [68–70]. AFLP is a great valued technique for gene mapping studies due to their high abundance and random distribution throughout the genome [64]. A linkage map of cotton was developed using the AFLP and RAPD markers [71]. AFLP markers have also been used for analyzing the genetic diversity [17, 72] and map saturation in cotton [19, 73].

#### 2.2.4. Microsatellites or Simple Sequence Repeats, SSR

These are di-, tri-, tetra- or pentatandom repeats of nucleotide, scattered abundantly in both noncoding and coding regions of a genome [29, 47]. Microsatellites are created from sphere where variants of repetitive DNA sequence are previously overrepresented [74]. The loci of these markers are highly transferable about 50% across species [75]. For SSRs analysis forward and reverse primers are employed in PCR reaction that anneal to the template DNA at the 5′ and 3′ ends. Short repetitive DNA sequences furnish the basis for multi allelic, codominant PCR based molecular marker and found more polymorphic as compare to other DNA markers [27, 47].

Due to their greater polymorphism, SSRs are considered as an important marker system in fingerprinting, analysis of genetic diversity, molecular mapping and marker assisted selection [76]. The availability of SSR markers in the cotton genome make them useful in study of genetic diversity [20]. Furthermore, over 1000 SSR primers have been designed from available cotton DNA sequences in genomic libraries [77]. Cotton Gen database is the largest repository for the SSR markers and their mapping information (http://www.cottongen.org/find/mapped_markers).

*(1) EST-SSRs.* SSR markers obtained from ESTs (expressed sequence tags) are present in sequences of functional gene and directly associated with transcribed parts of DNA [78]. About 1–5% of the ESTs in different species of plants have SSRs of suitable length for development of markers [79]. As compare to genomic SSRs, EST-SSR markers have greater potential for transferability between the species [80]. EST-SSRs also have a greater possibility of being functionally linked with variations in gene expression than genomic SSRs [81]. Rising number of ESTs for cotton helped in the recognition of SSRs domains from the ESTs by data mining methods. Recently, several EST-SSRs have been mapped in cotton [82–85]. However, EST-SSRs exhibit low level of polymorphism than conventional SSRs [86].

*(2) CAPS Microsatellites.* Cleaved amplified polymorphic sequence (CAPS) technique is actually the combination of RFLP and PCR [87], in which DNA fragments are amplified through PCR, followed by digestion with a restriction enzyme [88]. Subsequently, polymorphisms arise from the variation in the incidence of restriction sites of identified alleles are detected by gel electrophoresis [88]. CAPS microsatellite and CAPS are technically alike and use of microsatellite spheres and flanking regions as a template may have a decisive improvement over the well-known DNA markers in crop species. CAPS microsatellites change the monomorphic markers into polymorphic markers which mostly inherited in codominant way [89] and exhibit high polymorphism between strongly related genotypes. Any base substitutions can be identified by CAPS microsatellites as the polymorphism exhibited by this technique is based on the sequence dissimilarities in the flanking regions aside from the microsatellite spheres. These markers also assist in the analysis of composite traits by gene mapping and propose the opportunity of identifying markers by physiological and biochemical characteristics of their gene products [86]. However, the CAPS markers are only developed where mutations create a recognition site for restriction enzyme [87].

### 2.3. Sequence Based DNA Markers

#### 2.3.1. Single Nucleotide Polymorphism

Variations of single nucleotide (A, T, C, G) in sequence of individual genome are known as single nucleotide polymorphism or SNPs [26]. These may occur in the noncoding, coding and intergenic regions of the genome, so allowing the detection of the genes due to the variations in the sequences of nucleotides [26, 90] and these are either nonsynonymous or synonymous within the coding regions of the genome. Synonymous changes can alter mRNA splicing that result the changes in the phenotype of an individual [91].

SNP markers are important tool for linkage mapping, map based cloning and marker assisted selection due to the high level of polymorphism. The codominant nature of SNPs makes these markers able to distinguish the heterozygous and homozygous alleles [92]. Narrow genetic base and allotetraploid genome has made the discovery of SNPs difficult in cotton [93]. Recently, use of high throughput sequencing techniques have made it possible to detect great numbers of SNP markers [94, 95], including organisms with limited molecular studies [96, 97] and organisms with slight genetic variation such as cotton [85]. In cotton, many researches have been conducted to observe diversity, characterization and mapping of SNPs in the nucleotide sequence of* Gossypium *genome [98, 99]. Recently, an international collaborative effort has developed 70 K SNP chip based on Illumina Infinium genotyping assay (Unpublished data; http://www.cottongen.org/node/1287616). This high-throughput genotyping assay will be a resource that will be used globally by public and private breeders, geneticists, and other researchers to enhance cotton genetic analysis, breeding, genome sequence assembly, and many other uses. Similarly, Gene Chip cotton genome array comprising of 239777 probe sets representing 21485 cotton transcripts has been developed and under validation step before commercially available by Affymetrix (http://www.affymetrix.com/products_services/arrays/specific/cotton.affx). The sequences used for SNP chip development were selected from GenBank, dbEST and RefSeq contributed by the collaborators globally. These high-throughput technologies will be helpful for fine mapping and subsequent gene discovery for important economic traits in cotton. Additionally, these resources will provide foundations to initiate genomic selection studies in cotton, ultimately enhancing genetic gain from breeding.

#### 2.3.2. Genotyping by Sequencing, GBS

Genotyping-by-sequencing (GBS) is a technique that simultaneously detects and genotypes the SNPs in a genome [100]. GBS was developed as a simple but strong access for reducing complexity in complex genomes [101]. The development of the GBS library is very simple. The original GBS method used a single restriction enzyme to capture the genomic sequence between restriction sites [101].

The choice of restriction enzyme is a crucial factor in GBS for covering the repetitive regions in the genomes. In the original GBS approach used in maize and barley, one restriction enzyme (RE) “*Ape*KI” was used which is methylation-sensitive to reduce the complexity of the genome and to choose hypomethylated sphere of the genome for sequencing [101]. A modified GBS approach was also developed in which two enzymes and a Y-adapter were used to generate “uniform” GBS libraries where Adapter 1 and Adapter 2 were on opposite ends of every fragment [102]. GBS is a multiple approach that can discover thousands of SNPs in an experiment and suitable for population studies, genomic selection, genetic mapping, germplasm characterization, and other breeding applications in different organisms [101–103]. GBS technique can also be employed in species of plants that do not have available reference genome. In these cases, the sequence tags can be deal as dominant markers for analysis [100].

## 3. Overview of Marker Based Crop Improvement Efforts

### 3.1. Genetic Diversity in Cotton

The success of any breeding program mainly depends on the availability of the genetic diversity in the germplasm resources. Understanding of the genetic relationships among plant genotypes is significant to know the complexity of available germplasm, to discover the differences in available genotypes and to build up useful conservation plans [104]. Thus, evaluation based on the molecular markers can give valuable insight into the genetic structure of a plant population, which helps in the development of new varieties [105]. The genetic diversity studies in cotton germplasm using different marker technologies are summarized in Table 2. A narrow genetic base is reported in cotton by several workers using different molecular markers [23, 38, 49, 106, 107].

RAPD and ISSR techniques have been utilized to analyze genetic diversity and hybridization and for the incident of somaclonal variations in various crops involving cotton [108–112]. Five prominent studies were conducted to evaluate genetic diversity using RAPD markers during 90s. Genetic diversity of 16 elite homozygous genotypes obtained from the inter-specific hybridization was studied using 80 RAPD markers [23]. RAPD markers were used to differentiate the* G. hirsutum* lines from the* G. arboretum *[113]. Similarly, 25 short duration genotypes of cotton were analyzed using arbitrary primers [114]. Later, [115] studied genetic diversity of 31* Gossypium* species, 3 subspecies, and 1 interspecific hybrid using 45 RAPD primers and the results showed that genetic relationship of many species is related to the center of origin. Recently, genetic diversity in 18 cotton genotypes of Pakistan studied by 5 RAPD primers showed that two diverse genotypes of cotton (CIM-240 and CIM-443) have resistance against cotton leaf curl virus [116].

AFLP technique was also used to distinguish the differences among diploid and tetraploid species of cotton by utilizing the variations in ribosomal RNA genes [106]. The genetic diversity between the upland cotton, wild species (*G. raimondii, G. thurberii,* and* G. sturtianum*) and their BC_3_ progenies was evaluated using AFLP markers [117]. Intra- and interspecific relatedness of the* G. barbadense, G. arboreum, G. raimondii,* and* G. hirsutum* are determined by AFLPs which demonstrated its usefulness for genetic relatedness across wide range of species [17]. The relationship between the parents and four day neutral backcross generations of cotton was determined using 43 AFLP markers [68]. Comparative study was conducted to evaluate AFLP and RAPD techniques using 16 diploid cotton genotypes and it was concluded that AFLP markers are more efficient for polymorphism detection and for analyzing of genetic diversity as compared to RAPDs [72]. Similarly, genetic diversity of 26 Tanzanian cotton genotypes (*Gossypium hirsutum *L.) was studied using the AFLP markers [66]. The results of this study indicated the high values of genetic similarity which show the lower genetic diversity among Tanzanian cotton cultivars. Reference [65] mapped 98 AFLP markers and assigned 22 distinctive chromosomal positions using cytogenetic deletion stocks. Mapping information enhanced the utilization of AFLPs and can be used to saturate the existing marker frequency over different chromosomes.

In cotton, SSRs are considered as a new class of DNA markers which hastened cotton genetic diversity and mapping studies [27] and are important source to observe the transcribed genes [118]. There are multiple reports about using the SSR markers for genetic diversity. Reference [119] identified 71 SSR loci with 65 primer pairs and placed them on distinctive chromosomes of cotton. Genetic diversity among U.S. and Australian cultivars, and day neutral lines of* G. hirsutum* was also analyzed by SSR markers [120].

Further saturation of SSR markers was extended by addition of 204 markers which exhibited 261 segregating bands giving rise to 233 mapped loci in cotton [77]. Interspecific polymorphism between* G. barbadense* and* G. hirsutum* was also studied using SSR markers and results showed that polymorphism between species was high but it was low within species [121]. Reference [122] developed new SSR markers, analyzed the status of 23 chromosomes and found that the inter loci distance was 4.9 cM. Diversity among 52 different* G. hirsutum* cultivars was studied by 31 SSR primer pairs and successfully discriminated the 52 cultivars through broader allelic coverage [123]. Similarly, genetic diversity of 43 upland cotton varieties [124], 56 sea-island cotton accessions [125], 19* Bt* cotton genotypes [107], 50 representative Pakistani genotypes [104], and 193 upland cotton cultivars [126] were evaluated using 36, 237, 104, 70, and 448 SSR markers, respectively. SSRs have also been used to assess the genetic purity of the cotton hybrids [127] and demonstrated as an effective tool for hybrid identification.

Recent developments in next generation sequencing (NGS) and RNA-seq technology have generated high-throughput sequence data which facilitated the identification of SNPs as effective and highly saturated markers for genetic studies in cotton. Genetic variations within and between the different species of cotton have been characterized by 1000 SNPs and 279 In-Dels from the 270 and 92 loci segregating in* G. barbadense *and* G. hirsutum* to provide mapped molecular markers for crosses within species and introgression of foreign germplasm in cotton [99]. A genome reduction experiment based on the restriction site conservation (GR-RSC) and previously generated assembly of express sequence tags (ESTs) were used to discover the SNPs in 4 accessions of* G. hirsutum* and* G. barbadense*. A total 11,834 and 1,679 non-genic and 4,327 genic SNPs were identified in the GR-RSC and EST assemblies using highly conservation parameters. The KASPer assays were used to target the 1,052 (704 nongenic and 348 genic) genome specific SNPs between the* G. hirsutum* accessions [93]. The assay then tested for the Mendelian segregation ratio in the F_2_ population derived from a cross of upland cotton (*G. hirsutum*) cultivars.

### 3.2. QTLs Mapping for Important Economic Traits in Cotton

The regions in genomes to have genes linked with a quantitative trait are known as quantitative trait loci, QTLs [128], and the process of developing linkage maps and performing QTL analysis is referred to as QTL mapping [129, 130]. QTL analysis stands on the principal of identifying a connection among phenotype and genotype of markers [128]. The QTLs identified in cotton germplasm using different marker technologies are summarized in Table 3.

RFLPs have been widely used to map genes of economic interest in cotton. Previously, RFLP map of* G. hirsutum *and* G. barbadense *was used to map 14 QTLs for fiber related traits [131]. Similarly, genes influencing density of stem and leaf trichomes [132], high gossypol plant, and low seed gossypol contents [117] were confined by RFLP markers. Reference [131] developed an RFLP map of 261 markers distributed among 26 linkage groups using F_2_ plants from an interspecific cross. Another genetic linkage map was developed using RFLP markers, and 26 QTLs were recognized for agronomic and fiber quality traits [41]. Later on RFLP based QTL mapping was extended to leaf chlorophyll contents [133]. Backcross population of* G. hirsutum* and* G. barbadense *was used to map 28, 9, and 8 QTLs for fiber length, length uniformity, and short fiber contents, respectively, using the 262 RFLP markers [134].

RAPDs have also been widely used for QTL mapping in cotton; however, lack of reproducibility and unknown chromosomal positions remained main disadvantages which restricted the use of RAPDs in advanced studies. Reference [135] used 85 RAPD markers and identified 13 QTLs associated to the fiber quality in the F_2_ population derived from the* G. hirsutum* and* G. barbadense *cross. There are numerous studies on using the RAPDs for QTL mapping along with other molecular markers (Table 3). An extensive SSR genotyping was conducted over F_2_ populations from 3 diverse upland cotton genotypes using 1378 markers and 39 fiber related QTLs were identified [136]. Recombinant inbred lines (RILs) are also important mapping populations and several QTLs related to plant architecture [137], yield [3], and fiber quality [19] have been identified in upland cotton using RILs. About 31 QTLs linked to the yield and fiber quality traits are detected by wide array of SSR and EST SSR markers (6123) in 4 way cross populations developed from the 4 inbred lines of* G. hirsutum* [138]. A genetic linkage map of the tetraploid cotton was developed using 1601 pairs of SSR and 247 SNP markers [139]. The genetic map consisted of the 2072 loci covering 3380 cm of the cotton genome. Two F_2_ populations were generated by the crosses of upland cotton cultivars and 4083 SSR markers were used for QTL analysis, which detect 54 QTLs linked to early maturity [140].

A total of 144 primer combinations of AFLPs and 150 of SSRs were used to detect 28 QTLs related to the fiber traits [141]. To know the significant threshold for the LR statistics, permutation tests were carried out after which 7 QTLs remain significant. RIL lines developed from the intraspecific cross of upland cotton are used to detect the 12 epistatic and 4 main QTLs related to the plant architectural traits by 2130 SSR, 2 RAPD and 1 SRAP markers [137].

Conclusively, huge arrays of QTLs have been identified using multiple molecular marker technologies. Description of stable QTL from diverse generations, common QTL from various populations and homologous QTLs raises the information on the genetic base. Information about distribution of important QTLs in the genome of cotton is very important and promises the future strategy for marker assisted breeding. Cotton Gen serves as an important database for such information and currently this database has 988 QTLs for 25 different traits (http://www.cottongen.org/data/qtl) which can be surveyed according to objectivity.

### 3.3. Genome Wide Association Studies (GWAS) in Cotton

Association mapping, also known as linkage disequilibrium (LD) mapping, has appeared as a tool to determine the variation in complex traits using historical and evolutionary recombination actions at the population level [142]. In association mapping nonstructured populations are phenotyped and genotyped to identify the trait associated with marker [143]. This results into capture of wider recombination and higher resolution mapping as compared to linkage mapping [144]. The applications of association mapping for cotton assist extensive employment of natural genetic diversity conserved within the worldwide collections of cotton germplasm [145], as in other plant germplasm resources. Turning the efforts of gene-tagging from biparental QTL mapping to LD-based association study promise the productive employment of* ex situ* conserved genetic diversity of global germplasm resources of cotton [10]. The cotton genome may need few numbers of markers for productive associating mapping of complex traits, which is also reported for other crops [146]. Regarding the tetraploid genome of cotton with a total recombination length of about 5,200 cm and an average 400 kb per cm [22], the LD block sizes of ~5-6 cm distance is sufficient to conduct an association mapping of different traits that would require a maximum of ~1,000 polymorphic markers for successful and reliable association mapping [147]. Extent of genome-wide LD and association mapping of fiber quality traits were reported using 95 SSR markers in 285 exotic accessions of* G. hirsutum *comprised of 208 landraces and 77 varieties [10]. Similarly, LD-based association mapping was conducted for fiber quality traits in 335* G. hirsutum* germplasm using 202 SSR markers [147]. Progress in genome sequencing technology provides an opportunity to produce large size genotypic data, which supports association mapping over QTL mapping and because of this association mapping is becoming more common [148].

### 3.4. Marker Assisted Selection (MAS) in Cotton

Marker assisted selection (MAS) is a procedure by which a phenotype is selected on the basis of genotype of a marker [128]. Selecting the plants in the segregating population that have the suitable genes combinations is the important component of plant breeding [149]. Once the markers tightly linked to the genes have been detected, breeders may use particular DNA marker to identify the plants carry the genes [150]. The effectiveness and cost of MAS are influenced by the marker technique; therefore, it must be selected carefully [151]. During the past two decades, RAPDs techniques have been used for MAS for getting the glanded plants and glandless seeds in the interspecific population of* G. sturtianum *and other species [152]. It was exposed that DNA markers connected to the major QTL (QTLFS1) for fiber strength could be utilized in MAS to increase fiber strength of commercial varieties in segregating populations [153]. Some RAPD markers were developed into locus specific sequence characterized amplified region (SCAR) markers to screen the BC_1_F_4_ upland cotton. For example, SCAR 1920 marker for the major fiber strength QTL was developed and has been used for selecting desirable genotypes [154]. Screening of the SNPs which are mapped on chromosome 10 recognized extra 3 SNP markers that were associated with blue disease resistance gene (*Cbd*) which were employed to efficiently characterize a trait allowing MAS for strong levels of blue disease resistance in cotton breeding programs [155].

## 4. Cotton Draft Genome and Its Implication

The increasing information of DNA sequencing allows the discovery of genes and molecular markers associated with different traits, opening new avenues for crop improvement [148]. Sequencing of DNA promises to display the spectrum of diversity in the genus* Gossypium*. The tetraploid cotton species (2*n* = 4*x* = 52), such as* G. hirsutum *and* G. barbadense, *are thought to have developed by an allopolyploidization that happened nearly 1-2 million years ago, in which a D-genome species is pollen parent and species of an A-genome is maternal parent [12, 156]. It is essential to have a basic awareness of the structure of the component genomes to understand the cultivated polyploid genomes, their evolution, and interaction between their subgenomes. Toward the long-term aim of characterizing the diversity among cotton genomes, the cotton geneticists have prioritized the D genome progenitor* G. raimondii* for complete sequencing.* G. raimondii *has a ~880 Mb genome [157], the smallest genome in the genus* Gossypium* at ~60% of the size of diploid A-genome and 40% of the tetraploids [158]. A physical map of* G. raimondii *genome was assembled and several evidences referred that the* G. raimondii* genome is composed of two different qualitative components, one that is gene-rich and another that is repeat-rich [158]. About 40,976 protein coding genes, and 2,355 syntenic blocks identified in the genome of* G. raimondii *[159]. Similarly, the sequencing and assembling of* G. arboreum* genome depicted that 68.5% of the genome is covered by repetitive DNA sequences and about 41,330 protein-coding genes were predicted in the genome of* G. arboreum* [160].

## 5. Future Prospects

Cotton is a major source of foreign exchange for many countries around the globe; therefore, major focus remains the enhancement of yield and quality of fiber. This challenge can be accomplished by introducing new alleles from wild species [161, 162] and use of modern molecular technologies helping in increasing genetic gain of economic traits. In this scenario, it is believed that sequencing of the* G. raimondii* [159] and* G. arboreum* [160] draft genomes will facilitate the gene discovery of important traits. These genome resources can also be used for discovery of high-throughput marker platforms like Select SNP arrays. These high-throughput DNA markers will be helpful in recognizing the cotton genotypes carrying desired characters and was successfully used not only to study the genetic diversity but to develop linkage maps and mapping agronomic traits [12, 20], which are necessary for acceleration of varietal development. Although the QTL mapping for the various traits, that is, fiber yield and quality [131], drought tolerance [133], disease resistance [163, 164], and pests resistance [165] have been accomplished in cotton but these may not be helpful to clone causal genes due to lower marker densities. In general, the choice of a molecular marker technique is based on reliability, statistical power, and level of polymorphisms. Since their invention they are being continuously modified for improved utility to solve many problems and to bring forth automation. When these markers techniques reach a greater degree of automation then it will be suitable to use DNA markers directing to a new “Green Revolution” in the agricultural world.

Presently, the enormous development of more efficient DNA markers will go on in the future, because they can serve as an important tool for the plant breeders and geneticists to develop the cultivars of cotton that are demanded by the society. It has been proposed that SNPs marker will have large influence on MAS and mapping studies in future due to high abundance and development of sophisticated detection system [195]. GBS will clearly become the marker genotyping platform in coming years. So the development of novel markers such as GBS and SNPs and the accessibility of modern technologies such as DNA Chips and microarrays hasten genome mapping and subsequent gene discovery in the cotton for efficient cotton varietal development.

## Figures and Tables

**Table 1:** Comparison of marker systems in cotton.

Marker	Template DNA quantity	Template DNA quality	Genetics	Cost	Reliability	Reference
RFLPs	High	High	Codominant	High	High	[26, 47, 166]
RAPDs	Low	High	Dominant	Low	Low	[27, 128]
ISSRs	Low	Medium	Dominant	Low	Medium	[36, 47]
SSRs	Low	Moderate	Codominant	Low	High	[27, 166]
AFLPs	Medium	Moderate	Dominant	Moderate	High	[47, 100, 128]
SNPs	Low	High	Codominant	Low	High	[92, 166, 167]
GBS	Low	High	—	Low to moderate	High	[100, 168]

**Table 2:** An overview of genetic diversity studies in cotton using molecular markers.

Marker	Country	Population type	Size	References
RAPDs	USA	16 near-homozygous elite cotton genotypes	80 RAPD primers	[23]
	Pakistan	31 *Gossypium* species, 3 subspecies and 1 interspecific hybrid	45 RAPD primers	[115]
	USA	10 varieties of Upland cotton	86 RAPD primers	[32]
	Pakistan	30 cultivars of *G. arboretum *	50 RAPD primers	[169]
	Iran	13 F_1_ and F_2_ cotton genotypes (*Gossypium hirsutum*) obtained by crossing	30 RAPD primers	[170]
	India	Intraspecific cotton F_1_ hybrid and its parents	20 RAPD primers	[171]
	Iran	11 genotypes of *G. hirsutum* including 6 F_2_ progenies	40 RAPD primers	[172]

SSRs	USA	24 cultivars of *G. hirsutum *	88 SSR primers	[173]
	China	39 accessions of* G. arboreum *	358 SSR primers	[174]
	China	108 accessions of Asiatic cotton and 1 *G. herbaceum *	60 SSR primers	[175]
	China	43 sources of Upland cotton germplasm	36 SSR primers	[124]
	France	47 genotypes of tetraploid cotton	320 SSR primers	[176]
	USA	96 accessions of* G. arboreum *	115 Genomic and EST-SSRs	[177]
	Pakistan	8 accessions of *G. hirsutum *	32 SSR primers	[178]
	China	59 genotypes of *G. hirsutum *	40 EST-SSR primers	[179]
	Greece	29 genotypes of *G. hirsutum* and an interspecific hybrid	12 SSR primer pairs	[180]
	China	56 accessions of Sea island cotton	237 SSR primers	[125]
	Egypt	28 genotypes of Egyptian cotton	6 SSR and 5 EST-SSR primer	[2]
	Pakistan	19 genotypes of *Bt* cotton	104 SSR primers	[107]
	USA	193 cultivars of Upland cotton	448 SSR primers	[126]
	USA	378 accessions of *G. hirsutum* and 3 of *G. barbadense *	120 SSR primers	[181]
	Iran	11 genotypes of *G. hirsutum* including 6 F_2_ progenies	4 SSR primers	[172]

AFLPs	USA	11 accessions of *G. hirsutum *and 2 from each of *G. arboreum*, *G. herbaceum* and *G. barbadense *	4 *EcoRI-MseI* primers for AFLP	[106]
	Belgium	Upland cotton, wild species (*G. raimondii and G. thurbrii*) and their BC_3_ progenies	5 AFLP primers	[117]
	USA	3 diploid species of *Gossypium*, 3 *G. barbadense* cultivars and 43 *G. hirsutum *accessions	20 AFLP primers	[13]
	USA	29 accessions from five species of the genus* Gossypium *	16 AFLP primers	[17]
	India	24 lines of *G. hirsutum* L.	6 AFLP primers	[182]
	Norway	131 accessions of *G. barbadanse *	8 AFLP primers	[183]

ISSRs	Iran	13 F_1_ and F_2_ cotton genotypes (*Gossypium hirsutum*)	8 ISSR primers	[170]
	Egypt	28 Egyptian cotton genotypes	5 ISSR primers	[2]
	India	Intraspecific cotton F_1_ hybrid and its parents	19 ISSR primers	[171]
	Iran	11 genotypes of *G. hirsutum* including 6 F_2_ progenies	20 ISSR primers	[172]

SNPs	Pakistan	2 cultivars of tetraploid cotton	Primers correspond to *FIF1* gene	[184]
	USA	24 lines of cotton	270 SNP loci and 92 Indel	[99]

**Table 3:** An overview of QTL studies in cotton.

Traits	Descriptor	Population	Size	Marker (number and type)	QTLs number	Reference
Fiber quality	FS, FL and FF	F_2_	171	RFLPs and 85 RAPDs	13	[135]
	FS	F_2_	186	217 SSRs, 800 RAPDs UBC and 1040 OPERON	2	[153]
	LY, LP, SW, NS, UQ, SF, FL, FE, FT, FF and IF	F_2_	120	144 AFLPs, RFLPs and 150 SSRs	28	[141]
	FS, FE, FL, FU, LP and FF	F_2_	117	290 SSRs and 9 AFLPs	16	[19]
	FF	BC_3_F_2_	3,662	262 RFLPs	41	[185]
	FL, FLU and SFC	BC_3_F_2_	3,662	262 RFLPs	45	[134]
	FS, FE, FU, FL and FF	RIL's	270	7508 SSRs, 384 SRAPs and 740 IT-ISJs	13	[186]
	FL, FS, FF and FE	F_2_	—	1378 SSRs	39	[136]
	FS, FL, FF, FMT, FE and SFI	RIL's	180	4106 SSRs, AFLPs, RAPDs and SRAPs	48	[187]
	FE, FL, FS, FF and FU	CP	172	16052 SSRs	63	[188]

Fiber and agronomical	SCY, LY, LP, BW, SI, FMT, PER, WF, WT, FF, FL, FE and FS	RIL's	188	141 SSRs	56	[189]

Yield and fiber	FS, FL, FF, FE, LP, SI, NB, SCY and LY	RIL's	258	2131 SSRs	53	[190]
	NB, BW, SI, LP, LI, SCY, LY, FL, FS, FF, FE and FU	4WC and inbred lines	280	6123 SSRs and EST-SSRs	31	[138]
	SCY, LY, NB, BW, LP, SI, LI and FBN	RIL's and IF2	180	2675 EST-SSRs	111	[191]
	PH, FBN, BW, LP, LI, SI, LY, FL, FS, FE, FF and FU	*G. hirsutum* accessions	81	121 SSRs	180	[3]
	LI, SI, LY, SCY, NSB and FS	F_2_	69	834 SSRs, 437 SRAPs, 107 RAPDs and 16 REMAPs	52	[192]

Morphological	LBNO, SL1, L1, W1, L2, W2, L3 and W3	F_2_	180	261 RFLPs	62	[193]
	EM	F_2_ and F_3_	—	4083 SSRs	54	[140]
	NFB	F_2_	251	1165 SSRs	5	[194]

Plant architectural	PH, FBL, FBN, FBA, FBL/PH and NMUB	RIL's	180	2130 SSRs, 2 RAPDs and 1 SRAP	16	[137]

NB: number of bolls per plant, BW: boll weight, SI: seed index, LP: lint percent, LI: lint index, SI: seed index, SCY: seed cotton yield per plant, LY: lint yield per plant, FL: fiber length, FS: fiber strength, FE: fiber elongation, FU: fiber uniformity ratio, FY: fiber yellowness, FF: fiber fineness, FMT: fiber maturity, PH: plant height, FBL: fruit branch length, FBN: fruit branch number, FBA: fruit branch angle, FLU: fiber length uniformity, SFC: short fiber content, FR: fiber reflectance, SW: seed weight, NS: number of seeds per plant, UQ: upper quartile length, SF: short fiber content, FT: fiber tenacity, IF: immature fiber content, SFI: short fiber index, NSB: number of seeds per boll, EM: early maturity, NMUB: lower, middle, and upside boll number, NFB: node to first fruiting branch, LBNO: lobe numbers, SL1: sublobe number on the main lobe, L1: main-lobe length, W1: main-lobe width, L2: second-lobe length, W2: second-lobe width, L3: third-lobe length, W3: third-lobe length, PER: perimeter, WF: weight fitness, WT: wall thickness, FBL/PH: ratio of fruit branch length to plant height, RIL's: recombinant inbred lines, IF2: immortalized F2s, 4WC: four way cross, CP: composite cross, and BC_3_F_2_ = backcross families.
